# Update on diffuse large B-cell lymphoma: highlights from the 2022 ASCO Annual Meeting

**DOI:** 10.20892/j.issn.2095-3941.2022.0403

**Published:** 2022-08-30

**Authors:** Minghan Qiu, Shan Wu, Xinrui Chen, Huaqing Wang

**Affiliations:** 1 Department of Oncology, Tianjin Union Medical Center, Nankai University, Tianjin 300191, China; 2 The Institute of Translational Medicine, Tianjin Union Medical Center, Nankai University, Tianjin 300121, China

Diffuse large B-cell lymphoma (DLBCL) is the most common non-Hodgkin’s lymphoma (NHL) and has high heterogeneity. Approximately 30%–50% of patients develop relapsed/refractory (R/R) disease, which remains a major cause of mortality^1–3^. In recent years, a variety of novel therapies have emerged, including bispecific T-cell engagers (BiTEs), antibody–drug conjugates (ADCs), chimeric antigen receptor T cells (CAR-T), and selective BTK inhibitors, which have provided effective treatment strategies for patients with DLBCL^1,4^. Recently, the 58th Annual Meeting of the American Society of Clinical Oncology (ASCO) was held in Chicago, presenting cutting-edge studies in DLBCL. Here, we discuss a selection of interesting data on this topic.

## Bispecific antibodies

BiTEs are bispecific antibodies designed to target both CD3 and tumor-specific antigens, which induce T-cell activity and promote tumor cell death^5^. Bispecific antibodies have the potential to revolutionize DLBCL therapy.

Glofitamab is a novel BiTE that exerts anti-tumor effects by binding both CD20 on B-cells and CD3ɛ on T-cells in a 2:1 configuration^6,7^. The results of a pivotal phase II extension study of glofitamab in patients with R/R DLBCL were orally presented by Australian researchers^8^. At a median follow-up of 12.6 months, 155 patients with DLBCL who had received at least 2 prior lines of therapy were included in the study. The objective response rate (ORR) and complete response rate (CRR) were 51.6% and 39.4%, respectively. The median progression-free survival (PFS) was 4.9 months, and the median duration of response was 18.4 months. Although cytokine release syndrome (CRS) occurred in 63% of patients, only 3.9% experienced grade 3 or higher effects.

Epcoritamab (Epco), another BiTE antibody targeting CD3/CD20, achieved an ORR of 68% and a CRR of 45% for R/R DLBCL in a previous study^9^. At the ASCO meeting, Falchi et al.^10^ reported an ORR of 96% and CRR of 68% for Epco + R-CHOP in 33 patients with untreated high-risk DLBCL. Other 2 phase 1/2 studies focused on patients with R/R DLBCL treated with Epco+GemOx and Epco+R-DHAX/C. The ORR values were 92% and 83%, and the CRR values were 60% and 61%, respectively^11,12^. All studies showed manageable safety. BiTEs showed promising efficacy not only in untreated patients but also in heavily pretreated patients, including those with prior exposure to CAR-T cells and/or with highly refractory DLBCL. Further studies focusing on BiTEs are proceeding in multiple countries, and BiTEs may become a new treatment option for DLBCL patients. Because BCL2 and TP53 mutations have been shown to be associated with poor prognosis in patients with DLBCL with R-CHOP treatment, BiTE therapy has high potential for those patients^3^.

## ADCs

ADCs contain a monoclonal antibody conjugated to a cytotoxic drug *via* a chemical linker. ADCs can selectively deliver cytotoxic drugs directly to target cancer cells^13^. Polatuzumab vedotin (Pola) is a CD79b-targeted ADC delivering the microtubule inhibitor monomethyl auristatin E (MMAE). Pola has shown promising efficacy for R/R DLBCL as monotherapy or combined with an anti-CD20 monoclonal antibody-containing regimen, thus resulting in ORRs of 13%–56%^14–16^. However, the CRRs have been unsatisfactory (0%–15%), thus prompting combination treatment with additional agents^14–16^. At the ASCO meeting, Lynch et al.^17^ presented preliminary results from an investigator-initiated trial for upfront treatment of aggressive B-cell NHLs. In this study, 18 patients received 6 cycles of Pola with dose-adjusted etoposide, cyclophosphamide, doxorubicin, and rituximab (Pola-DA-EPCH-R). The ORR was 88%, and the CRR was 24%^17^. However, 5 severe adverse events were observed, including one grade 5 sepsis/typhlitis, 3 febrile neutropenia, and one grade 3 perforated colonic diverticula. Other grade 3 adverse events (AEs) included hyperglycemia, oral mucositis, asymptomatic pulmonary embolism, abdominal pain, and hypokalemia^17^. These findings are similar to those for other Pola combination regimens presented at the annual meeting. Polatuzumab has shown favorable efficacy in patients with primary and R/R DLBCL. However, the increase in treatment-associated AEs may limit its clinical application^16,18^.

## CAR-T therapy

CAR-T therapy has changed the therapeutic landscape for several hematological malignancies with promising efficacy^19^. Axicabtagene ciloleucel, tisagenlecleucel, and lisoctagene maraleucel are autologous CAR-T-cell products targeting CD19, which have been approved by the U.S. Food & Drug Administration for the treatment of patients with DLBCL who have relapsed or have failed ≥2 line regimens, according to the JULIET, ZUMA-1, and TRANSCEND studies^20–23^. Patients with heavily pretreated DLBCL receiving CAR-T therapy have a median PFS of 5.9-6.8 months and a median overall survival (OS) of 11.1–21.1 months. The best ORRs of patients in these studies were approximately 52%–74%, and the CRRs were 40%–54%. The incidence of grade 3/4 CRS in the JULIET, ZUMA-1, and TRANSCEND studies was 10%, 22%, and 2%, respectively^20–23^. The results of these studies suggest the efficacy and safety of CAR-T cells as a therapeutic option for patients with R/R B-cell lymphoma. However, severe life-threatening toxicity, modest antitumor activity, antigen escape, and limited trafficking and tumor infiltration have restricted the clinical use of CAR-T therapy^24^. In a phase 1 study presented by US researchers at the 2022 ASCO Annual Meeting, anti-CD20 CAR-engineered allogeneic gamma delta (γδ) T cells (ADI-001) were used to treat patients with R/R B-cell lymphoma^25^. ADI-001 cells’ expression of histocompatibility complex independent γδ T-cell receptors enables them to directly recognize and bind tumor cell surface antigens, thus complementing CAR targeting while decreasing the risk of graft-*vs.*-host disease and the incidence of other AEs. In the 6 evaluable patients, the ORR was 67%, and all patients achieved complete remission after 28 days of follow-up. The AEs of special interest (SIAEs) were grade 1/2 CRS in 2 patients and grade 1 immune effector cell-associated neurotoxicity syndrome in 1 patient. Allogeneic CAR-T-cell therapy demonstrated promising efficacy and was well tolerated in patients with R/R DLBCL. Moreover, the development of allogeneic CAR T cells could potentially decrease the cost and increase access to this class of therapeutics^26^.

The RELIANCE study is a multicenter phase 2 trial of relmacabtagene autoleucel (relma-cel), an autologous CAR-T-cell product targeting CD19, in Chinese patients with R/R large B-cell lymphoma. At the 2022 ASCO Annual Meeting, Ying et al.^27^ presented the results of a 2-year follow-up of relma-cel in R/R DLBCL. Among 58 efficacy-evaluable patients, the best ORR and CRR were 77.6% and 53.5%, respectively. The 2-year PFS, duration of response, and OS rates were 38.3%, 38.1%, and 69.0%, respectively. The incidence of grade 3 or higher AEs was 72.9%, and hematological toxicity was the most common AE^27^.

Currently, CAR-T-cell therapy has significantly improved the prognosis of patients with R/R B-cell lymphoma, thus providing a new treatment strategy for patients unable to receive hematopoietic stem cell transplantation and whose disease progresses after multiple lines of therapy. Han et al.^28^ have developed a novel method to generate sufficient CAR-T cells from limited peripheral blood to treat B-cell malignancies, thereby providing an alternative to the traditional CAR-T cell generation method. However, limited sample sizes in clinical studies and severe toxicity remain barriers to developing effective CAR-T-cell therapies. More evidence is needed to evaluate the efficacy and safety of CAR-T-cell therapy^29^.

## Monoclonal antibodies

Targeting CD27 with monoclonal antibodies provides co-stimulation of immune cell activity^30^. Varlilumab is a novel agonist immunoglobulin G1 anti-CD27 antibody that mediates antitumor immunity and targets CD27, which is expressed on nearly all mature B-cell lymphomas^31^. Varlilumab has been demonstrated to cause T-cell activation and to demonstrate anti-tumor activity in preclinical models^30,32^. At the 2022 ASCO Annual Meeting, Villasboas presented the results of the DIAL study (NCI 10089), a randomized phase 2 trial of varlilumab combined with nivolumab in patients with R/R aggressive B-cell NHL^33^. A total of 53 patients enrolled in the study received nivolumab, either alone (group 1, *n* = 27) or combined with varlilumab (group 2, *n* = 26). The ORR, median OS, and PFS did not statistically differ between arms. AEs of grade 3 and above were observed in 8 (33.3%) and 7 (30.4%) patients in groups 1 and 2, respectively. Dual immunomodulatory therapy did not enhance anti-tumor activity in patients with aggressive B-NHL over that with nivolumab alone.

## Conclusion

The treatment modes for lymphoma are changing rapidly. Novel chemotherapy-free approaches, such as targeted therapy and immunotherapy, may lead to improved outcomes for patients with DLBCL and other B-cell lymphoma histologies. The results from the 2022 ASCO Annual Meeting indicated more possibilities for the treatment of lymphoma to provide patients with more therapeutic options.
